# Frequency of Hepatitis B, C, and Human Immunodeficiency Virus in Blood Donors

**DOI:** 10.7759/cureus.25978

**Published:** 2022-06-15

**Authors:** Rehana Ahmed, Mushkbar Fatima, Javeria Ashfaq, Syeda Faryal Tariq, Imran Naseer, Muhammad Asif, Munira Borhany

**Affiliations:** 1 Department of Hematology, National Institute of Blood Diseases and Bone Marrow Transplant, Karachi, PAK; 2 Department of Research and Development, National Institute of Blood Diseases and Bone Marrow Transplantation, Karachi, PAK; 3 Department of Clinical Hematology, National Institute of Blood Diseases and Bone Marrow Transplantation, Karachi, PAK; 4 Department of Hematology, National Institute of Blood Diseases and Bone Marrow Transplantation, Karachi, PAK

**Keywords:** immunodeficiency virus, blood transfusion, blood donors, hcv, hbv

## Abstract

Introduction: Blood donation is considered an important source of infection transmitted through transfusion, especially in developing countries like Pakistan.

Objective: To find out the frequency of seroprevalence of hepatitis B virus (HBV), hepatitis C virus (HCV), and human immunodeficiency virus (HIV) among blood donors in the blood bank.

Methods: A prospective cohort study was carried out on blood donors at the National Institute of Blood Disease and Bone Marrow Transplant, Karachi, during the period of January 1, 2019 to December 31, 2020. The descriptive statistical analysis to find out the percentages and frequencies was implemented using SPSS version 23 (IBM Corp., Armonk, NY).

Results: During the study duration, a total of 23,656 blood donors visited and donated blood, including 12,234 blood donors in the year 2019 and 11,422 blood donors in the year 2020. According to the analysis, only 1.4% of patients with HBV, 1.5% with HCV, and 0.03% were seropositive in the year 2020. In 2019, 1.6% HBV, 2.07% HCV, and 0.09% HIV blood donors were seropositive with a significant 0.00 *p*-value.

Conclusion: It is concluded that hepatitis C is the most commonly occurring in donors compared to HBV and HIV. HBV vaccines are available in Pakistan, which is why cases are fewer than HCV.

## Introduction

Donating blood seems to be a life-saving and rehabilitative technique that improves the lives of thousands of citizens annually. Per year, 90-100 million blood units are projected to be obtained around the globe [1]. The World Health Organization signed a statement in 2005 that committed to providing safe and enough medical supplies to patients [2]. Despite the advantages of blood donation, every donor is at the menace of contracting transfusion-communicable diseases, the most common of which are hepatitis B virus (HBV), hepatitis C virus (HCV), and human immunodeficiency virus (HIV). So, communicable diseases like HIV, HBV, and HCV are of special concern because of their lengthy infectivity and carrier status, as well as the fact that they induce a variety of serious illnesses that can lead to death [3,4]. HBV infects almost 350 million populations, HCV infects 200 million populations, and HIV infects 38 million populations [5,6]. According to the WHO's Worldwide Status Report on HIV and Hepatitis, HBV prevalence is expected to be 3.8% (3.0-5.5%), HCV prevalence is 0.8% (0.6-1.0%), and HIV prevalence is 0.7 percent (0.6-0.9 percent) [7,8]. Transfusion is most commonly used in developed countries to support sophisticated and complicated surgeries such as cardiac procedures, organ transplantation, and advanced trauma care, whereas it is primarily used in Sub-Saharan Africa to treat anemic patients with infectious diseases [9]. There is a broad range of bloodborne infections that can be transferred through the blood of healthy subjects and asymptomatic blood donors in developing countries [10]. Pakistan has a transfer rate of around 1.5 million people annually [11]. The hospital's blood banks consider the complete haemovigilance process (from source to recipient) but are not restricted to drawing blood, testing, cross-matching, and transfer. Numerous commercial blood banks are operating around the state with weak quality assurance, testing techniques, and preservation and donation standards. Several non-profit organizations, in addition to the business sector, provide donation services for certain demographic groups, such as sickle cell anemia individuals [12]. TTIs have become a major problem in Pakistan due to a lack of coordination among hospitals and other donation providers, jeopardizing the health of both donors and receivers. Several studies in developing and developed countries were conducted to determine the incidence of HBV, HCV, and HIV among blood donors [13]. The focus of this research was to determine the frequency of HBV, HCV, and HIV seroprevalence in blood donors who attended a care facility in Karachi. The incidence of transfusion-transmissible diseases among bone marrow donors will be tracked using this as a benchmark. This will contribute to the creation of policies aimed at improving the safety of blood transfusions.

## Materials and methods

A prospective cohort study was conducted at the National Institute of Blood Disease and Bone-marrow Transplantation, Karachi, Pakistan, during the period of 2019 to 2020.

A total of 23,656 blood donors (both exchange and volunteer) were evaluated, including 12,234 blood donors in the year 2019 and 11,422 blood donors in the year 2020. The Institutional Review Board (IRB) was consulted about the study. Healthy blood donors of either gender or age group of 18 to 50 years were selected for blood donation. A physical examination of each blood donor was performed. After that, clinical and medical history were taken to exclude patients having a positive history of hepatitis B or C or HIV or having any other severe disease. Patients with a normal body weight of >50 kg were included for blood donation. First time and repeat, as well as exchange and volunteer donors, were both included in the study. A 4 ml blood sample of each blood donor was obtained in an aseptic environment in a sterile container and screened for HBV, HCV, and HIV within two hours of blood sample collection. An automated enzyme immunoassay analyzer was used for blood screening. SPSS version 23 (IBM Corp., Armonk, NY) was used to implement the descriptive statistical analysis to find out the percentages and frequencies. A chi-square was also used.

## Results

**Figure 1:**
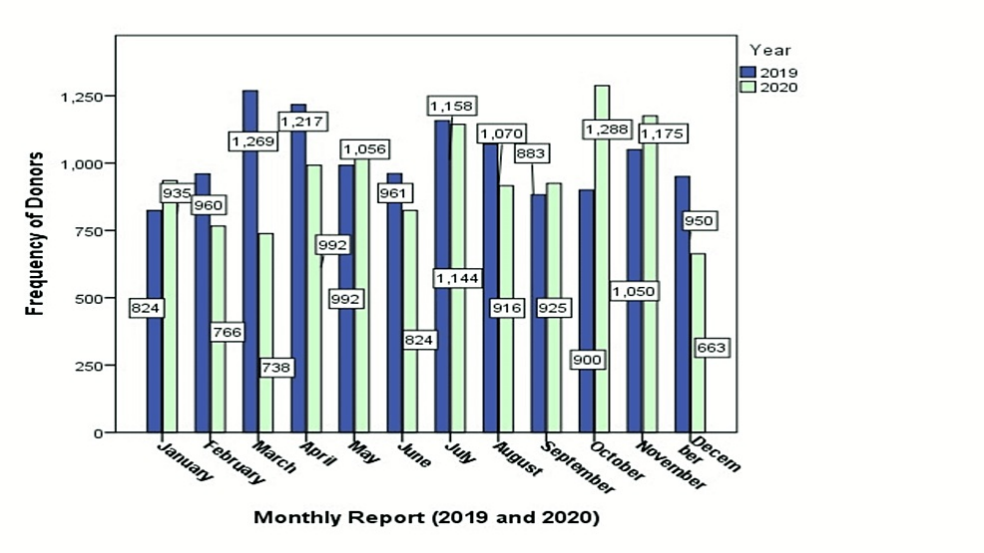
Frequency of screened blood donors during 2019 and 2020

In Figure 1, a graphical explanation of donors was presented. Twenty-three thousand six hundred fifty-six blood donors (both exchange and volunteer) have been evaluated, of which 12,234 blood donors were from the year 2019 and 11,422 blood donors from the year 2020 with a significant p-value of 0.00, and the monthly report shows that at least 824 individuals in January 2019 and 663 individuals in December 2020 donated their blood, while a maximum of 1269 individuals donated their blood in the month of March 2019 and 1288 in October 2020. More details have been presented below.

**Figure 2:**
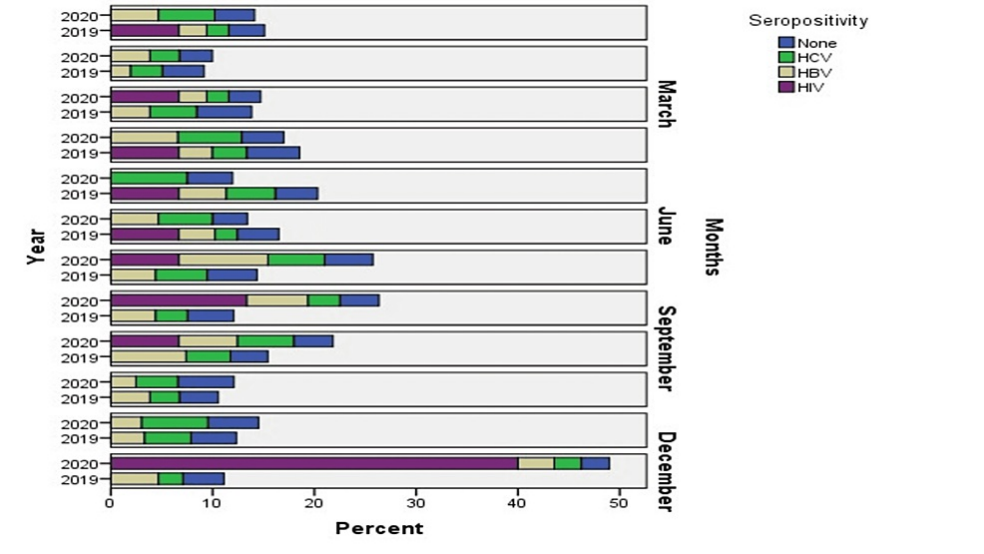
Explanation of seropositive blood donors during 2019 and 2020

**Figure 3:**
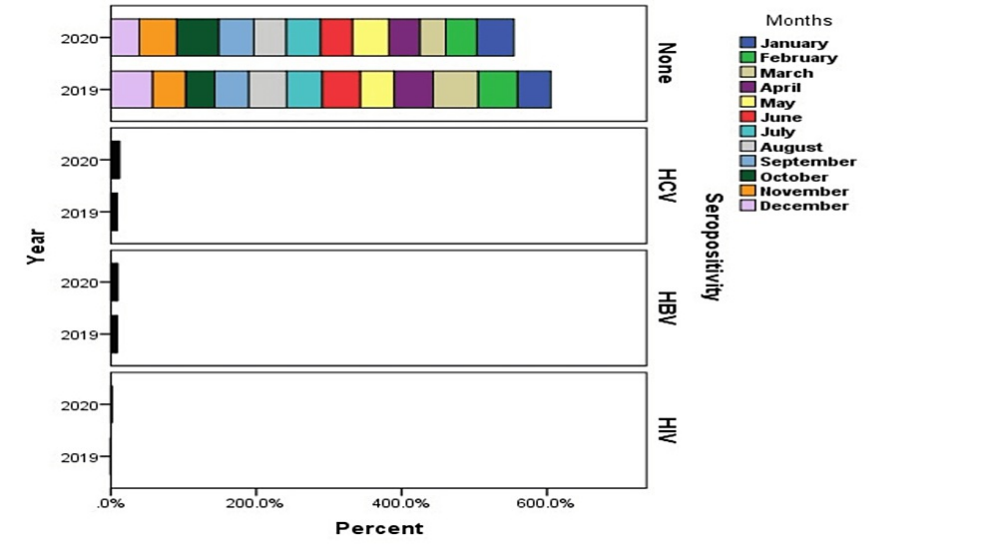
Graphical explanation of seropositive blood donors in each month of study

In Figures 2-3, a graphical explanation of the frequency of seropositivity of donors was presented. According to the analysis, in the year 2019, HIV was undetected in January, March, July, August, September, October, and November, and it was detected only in February, April, May, June, and December with 0.4%. While in 2020, HIV was undetected in January, February, April, May, June, October, and November, and it was detected in the months of March, July, August, September, and December in 0.4% of blood donors.

HBV was detected in blood donors who were screened for the whole 12 months of 2019 and 2020. In 2020, HBV was detected at 1.7% in January, 1.6% in February, 1.3% in March, 2.1% in April, 2% in May, 1.6% in June, 2.7% in July, 2.2% in August, and September, 9.6% in October, 1% in November, and 1.85% in December in blood donors. More details have been given in Figures 2-3.

In 2019, blood donors were also seropositive with a particular frequency, as presented in Figures 2-3. The frequencies of HCV seropositive blood donors were displayed as, for example, 1.09% in January, 1.3% in February, 1.5% in March and May, 1.1% in April, 0.8% in June, 1.6% in July, 1.2% in August, 1.9% in September, 1.3% in October, 1.7% in November, and 1.05% in December. In 2020, blood donors were also positive with particular frequency, e.g., 2.4% in January, 1.5% in February, 1.2% in March, 2.2% in April, 2.4% in May, 2.3% in June, 2.0% in July, 1.2% in August, 2.5% in September, 1.3% in October and November, and 1.6% in December. In Table 1, HBV, HCV, and HIV-positive blood donors have been presented. According to analysis in our study, only 1.4% of patients with HBV, 1.5% with HCV, and 0.03% were seropositive in the year 2020. In 2019, 1.6% HBV, 2.07% HCV, and 0.09% HIV blood donors were seropositive with a 0.00 p-value. More details are presented in Table 1.

**Table 1:** Complete seropositivity report of screened donors *Correlation is significant at the ≤0.05 level

Year of study	Seropositivity report				Total	P-value	Spearman correlation
	HBV (%)	HCV (%)	HIV (%)	None (not diagnosed with any disease)			
2020	175 (1.4)	177 (1.5)	4 (0.03)	11,878	12,234	0.00*	0.02*
2019	190 (1.6)	237 (2.07)	11 (0.09)	10,984	11,422		
Total	365	414	15	22,862	23,656		

## Discussion

Transfusion has been known to save millions of lives. One of the most serious risks associated with transfusion is infection from microorganisms present in blood and products. Transfusion operations in the nation are neither quality guaranteed or standardized as a result of poor administration and regulation. In all provinces, blood transfusion agencies, which regulate inspection, licensing, and data management, have been alerted, but they are still in their infancy. Among compensated blood donors, transfusion-transmitted illnesses are regularly identified. To reduce the danger of transfusion-transmitted illnesses, several affluent countries have banned the use of compensated blood donors. Until 1998, the Chinese government prohibited paid blood donations, and many blood banks now rely only on volunteer donations. This method has helped to reduce HCV prevalence in China's general population from 8.68% in 1990 to 3.2% in 2010 [14]. In 1998, India, Pakistan's neighbor, implemented a rule prohibiting all compensated donations, successfully reducing the occurrence of TTIs transmission [15]. In one local study [14], it was demonstrated that the prevalence of hepatitis B, hepatitis C, and human immunodeficiency viruses was found to be 1.84%, 1.7%, and 0.04%, respectively. These results were consistent with the current study. In our study, patients in 2020 were less prevalent as compared to 2019 because of the COVID-19 influence in the country. According to Anwar et al. [16], the proportion of HBsAg and anti-HCV was found to be 7.94% and 2.79%, respectively. The patients were seropositive in the year 2020. In 2019, 1.6% of HBV blood donors, 2.07% of HIV blood donors, and 0.09% of HIV blood donations were seropositive, all with 0.00 p-values. Hepatitis B cases seem lower than hepatitis C because vaccinations for hepatitis B are available in Pakistan. In Pakistan, where there is only a nascent culture of charity contributions, a large dependence on replacement, and no systematic screening, infection risks are higher since relative substitute donors are more likely than voluntary donors to spread transfusion-transmissible illnesses [17,18]. Different studies reported similar and different prevalences of HBV in their settings, such as Farooq et al. 2.13% [18], Jiskani et al. [19] 1.38%, Ahmed et al. [20] 1.5%, Ahmad et al. [21] 1.4%, Mohsenizadeh et al. [22] 0.19%, Abebe et al. [23] 3.06%, Jary et al. [24] 14.78%, and Tigabu et al. [25] 4.1%. Different studies reported similar and different prevalences of HCV in their settings, such as Jiskani et al. [19] 2.14%, Ahmed et al. [20] 0.5%, Ahmad et al. [21] 0.60%, Mohsenizadeh et al. [22] 0.07%, Abebe et al. [23] 0.64%, Jary et al. [24] 2.32%, and Tigabu et al. [25] 1.6%. Our study findings were similar to most of the studies, particularly those performed in Pakistan [14-16]. The prevalence of HCV, HBV, and HIV is continuously increasing due to blood transfusion, so blood screening is highly recommended before blood donation.

## Conclusions

It is concluded that hepatitis C most commonly occurs in donors rather than HBV and HIV. HBV vaccines are available in Pakistan, which is why cases are fewer than for HCV. The rate of infection, including HCV, HBV, and HIV, is high in the year 2020 as compared to the year 2019. The differences between healthcare and personal regular inspections, as well as the socioeconomic categories served by various institutions, make it difficult to reduce the prevalence of HBV, HCV, and HIV in blood donors and the general public. Appropriate procedures, such as rigorous donor screening and suitable guidelines for secure blood transfusion, as well as community studies, are required to minimize the chances of HBV, HCV, and HIV.
